# Editorial: Fundamental enrichment of ratio-based metrics in cardiology

**DOI:** 10.3389/fcvm.2022.1013194

**Published:** 2022-08-31

**Authors:** Peter L. M. Kerkhof, Qi Fu

**Affiliations:** ^1^Department of Radiology and Nuclear Medicine, Amsterdam University Medical Centers, Amsterdam, Netherlands; ^2^Institute for Exercise and Environmental Medicine at Texas Health Presbyterian Hospital Dallas, Internal Medicine at University of Texas Southwestern Medical Center, Dallas, TX, United States

**Keywords:** ratiology, ejection fraction, coronary flow reserve, global function index, augmentation index, exercise, aortic stenosis

## Introduction

Ratio theory has fascinated scholars since the early descriptions, then referred to as *anthyphairesis* (meaning reciprocal subtraction), and described in Book V of *Euclid's Elements* which theory was developed by Eudoxus, who lived around 350 BCE, half a century before Euclid (1).

Paired measurements such as systolic and diastolic blood pressure are often converted to yield their difference (i.e., pulse pressure), mean value, or ratio. For the ratio-based candidate the systolic pressure is divided by the diastolic pressure reading, yielding a dimensionless metric, also for the reciprocal (2). Such unitless ratios have acquired a prominent place in cardiology, although their isolated use may reflect an incomplete description of the data set that is actually available (3). The present theme-based collection of publications focuses on various traditional ratio-based applications, but now enriched with a more comprehensive interpretation of these dimensionless ratios. Essentially, the authors explore the impact of an associated companion metric based on the Pythagorean theorem. This companion is calculated for the data that is already available to derive the ratio, without the need to collect additional measurements. Only the combination of ratio and corresponding companion offers the full picture.

## Ratiology

The most popular index to evaluate ventricular performance concerns ejection fraction (EF). As not all investigators accept the universal validity of EF, appealing variants have been developed. Among them the global function index (GFI) which introduces the myocardial mass normalized to tissue density (to eliminate physical units for this term), and employs mean ventricular volume rather than filling volume (EDV). The paper by Diaz et al. nicely documents that from a mathematical point of view the GFI not only resembles EF, but also requires consideration of a companion metric corresponding with GFI. The same EF paradigm was evaluated in connection with ventriculo-arterial coupling (VAC) with special attention to the *golden ratio*. Antohi et al. found that VAC alone cannot adequately describe this coupling system. In their solid study these authors also document a major role for systolic time intervals, thus introducing the dimension of time besides the standard pressure and volume components.

Concerning the study of coronary flow reserve (CFR) in heart transplant patients, Cecere et al. conclude that the combined use of CFR and its companion provides more complete clinical and prognostic information on coronary microvasculopathy in these patients. This type of multiparametric analysis of coronary flow has also been extensively described for patients with elevated incidence of cardiovascular events due to a systemic inflammatory syndrome, permitting a more personalized characterization of these patients and being superior to that achieved by exclusive consideration of the ratio CFR (4).

In patients with aortic stenosis, clinical assessment of the disease severity may be underestimated if the echocardiographic image quality is poor. In addition, the accurate evaluation of aortic stenosis severity is difficult to assess when cardiac output is lower and left ventricular EF is reduced. To resolve these limitations, Mantha et al. demonstrated that the dimensionless index (DI) which is a simple ratio of the left ventricular outlet tract velocity-time integral (LVOT-VTI) to that of the aortic valve jet (AV-VTI), combined with its companion can be used. The authors suggest that the DI along with its companion can separate aortic stenosis severity with clarity and enables clinicians to assess the severity of AS without worrying about the magnitude of AV-VTI and LVOT-VTI if it is <0.25.

The importance of the companion metric, in addition to the ratio variable, was also demonstrated in the study by Fukuie et al. The authors examined systemic and aortic hemodynamics in healthy young men during mild-to-moderate cycling exercise in water and on land. They found that aortic augmentation index (AIx), calculated from aortic augmentation pressure and aortic pulse pressure, did not show a significant interaction between the environment (water vs. land) and exercise intensity, whereas the metric of AIx companion (AIxC) did. Specifically, exercise-induced increases in AIxC were significantly smaller in water than on land. These results suggest that the rise in arterial afterload with exercise is smaller in water than on land in healthy individuals. The Pythagorean theorem-based transformations could provide better insight into the underlying physiology. This notion is supported by the findings of another study by Fukuie et al. showing that AIx remained unchanged but AIxC decreased after 15-week head-out aquatic exercise training in middle-aged and elderly people. The authors also found that aortic systolic pressure, pulse pressure, the companion metric of aortic pulse pressure, and brachial-ankle and heart-ankle pulse wave velocities decreased after training.

## Information vs. knowledge

Finding a single metric that reflects the full information content embodied by two or more independent measurements refers to just another holy grail. Accumulation of details, especially when derived from primary variables by mathematical manipulation such as division, does not necessarily contribute to further understanding or promote knowledge. The process of gaining (further) insight requires availability of sound underlying concepts applied to available fundamental data. For example, ventricular pump action can be adequately characterized by the simultaneous measurement of pressure and volume, resulting in a graphical representation. This route permits calculation of stroke work and cardiac power output, each associated with sound physical dimensions. However, such knowledge is not embodied by EF, which construct only shows the outcome of a mathematical procedure (- yielding a bare number -) that we get when end-systolic volume is divided by EDV (5). Likewise GFI and VAC yield numbers that merely compete for relative impact, as all refer to interconnected algorithms based on ESV, EDV, their difference, ratio or average value. Moreover, GFI and VAC also each carry a corresponding companion, similar to EF. Note that the companion metric is not an alternative for the traditional ratio-based metric, but actually constitutes a mandatory complementary element that may not be neglected.

## Corollary

We conclude that incorporation of the documented companion metric offers incremental value to the study of cardiovascular (patho)physiology, and constitutes a fundamental enrichment for patient management in clinical practice.

## Author contributions

Both authors listed have made a substantial, direct, and intellectual contribution to the work and approved it for publication.

## Conflict of interest

The authors declare that the research was conducted in the absence of any commercial or financial relationships that could be construed as a potential conflict of interest.

## Publisher's note

All claims expressed in this article are solely those of the authors and do not necessarily represent those of their affiliated organizations, or those of the publisher, the editors and the reviewers. Any product that may be evaluated in this article, or claim that may be made by its manufacturer, is not guaranteed or endorsed by the publisher.
